# 

**Published:** 1909-03

**Authors:** 


					﻿Sixtv=fourth Year.
Vol. LXIV.
MARCH, 1909.
No. 8
BUFFALO
MEDICAL JOURNAL
Established "	AUSTIN FLINT >	Eh
EDITOR:
WILLIAM WARREN POTTER, M. D.
ASSISTANT EDITORS:
WILLIAM C. KRAUSS, M. D.
NELSON W. WILSON, M. D.
ASSOCIATE EDITORS:
JAMES WRIGHT PUTNAM, M. D. ERNEST WENDE, M. D.
JOHN PARMENTER, M. D.	JOHN A. MILLER, Ph. D.
MAUD J. FRYE, M. D.
				

